# Genetic Diversity of Neotropical *Myotis* (Chiroptera: Vespertilionidae) with an Emphasis on South American Species

**DOI:** 10.1371/journal.pone.0046578

**Published:** 2012-10-03

**Authors:** Roxanne J. Larsen, Michelle C. Knapp, Hugh H. Genoways, Faisal Ali Anwarali Khan, Peter A. Larsen, Don E. Wilson, Robert J. Baker

**Affiliations:** 1 Department of Biological Sciences, Texas Tech University, Lubbock, Texas, United States of America; 2 University of Nebraska State Museum, Lincoln, Nebraska, United States of America; 3 National Museum of Natural History, Smithsonian Institution, Washington, D. C., United States of America; Biodiversity Insitute of Ontario - University of Guelph, Canada

## Abstract

**Background:**

Cryptic morphological variation in the Chiropteran genus *Myotis* limits the understanding of species boundaries and species richness within the genus. Several authors have suggested that it is likely there are unrecognized species-level lineages of *Myotis* in the Neotropics. This study provides an assessment of the diversity in New World *Myotis* by analyzing cytochrome-*b* gene variation from an expansive sample ranging throughout North, Central, and South America. We provide baseline genetic data for researchers investigating phylogeographic and phylogenetic patterns of *Myotis* in these regions, with an emphasis on South America.

**Methodology and Principal Findings:**

Cytochrome-*b* sequences were generated and phylogenetically analyzed from 215 specimens, providing DNA sequence data for the most species of New World *Myotis* to date. Based on genetic data in our sample, and on comparisons with available DNA sequence data from GenBank, we estimate the number of species-level genetic lineages in South America alone to be at least 18, rather than the 15 species currently recognized.

**Conclusions:**

Our findings provide evidence that the perception of lower species richness in South American *Myotis* is largely due to a combination of cryptic morphological variation and insufficient sampling coverage in genetic-based systematic studies. A more accurate assessment of the level of diversity and species richness in New World *Myotis* is not only helpful for delimiting species boundaries, but also for understanding evolutionary processes within this globally distributed bat genus.

## Introduction

A well-defined perspective of the continental and insular biotic diversity of South and Central America remains difficult to resolve despite years of effort by systematists. An excellent example is the low species-level resolution and current state of knowledge of the mammalian genus *Myotis* (Chiroptera: Vespertilionidae). This poor resolution is largely due to limited genetic studies focused on species-level variation. Moreover, morphological variation within the genus is low (i.e., cryptic variation) [1], [2], [3] and limits the resolving power of classical morphological studies. For these reasons the taxonomy and systematics of New World *Myotis* is complex and often controversial [1], [4]–[12]. Recent taxonomic syntheses and publications focused on *Myotis* recognize ∼ 42 species distributed in the New World, with 26 species in North America, 11 in Central America, 15 in South America, and five in the Caribbean (Table 1) [12]–[16].

**Table 1:** Number of recognized species of New World *Myotis* based on general region and genetic distance value comparisons.

Region	Recognized	Endemic	≥2.0%	≥5.0%	n	Average pairwise divergence(Standard Error)
NA	26	18	25	13	41	13.73% (0.69)
CA	11	1	4	2	6	12.97% (0.78)
CB	5	3	4	4	15	8.13% (0.6)
SA	15	9	34	18	74	12.85% (0.66)
Total	42 (32*)	31	67	37	136	13.14% (1.26)

Number of recognized species and number of endemic species based on the current literature [12]–[16]. Note the number of genetic lineages by region and the total in each instance: all ≥2.0% genetic divergence and all ≥5.0% divergence (some species are found in more than one region). Asterisk indicates the number of currently recognized species represented in our dataset. n = number of individuals sequenced from the region. Average divergence value and standard error by region is also listed. Abbreviations: NA = North America, CA = Central America, CB = Caribbean, SA = South America.

The most recent DNA sequence-based overview of New World *Myotis* [3] examined one or a few individuals from 32 of the recognized species (those with available tissues). Here, we build on the findings of Stadelmann et al. [3] by generating DNA sequence data from an increased geographic sample of *Myotis* (especially in South America). Collectively, these genetic data serve to elucidate the diversity within *Myotis* (including cryptic species) and provide the basis for understanding the taxonomic boundaries of several wide-ranging species. Our baseline questions included: How many species-level lineages, based on cytochrome-*b*, are there in broadly defined geographic regions (i.e., North America, Central America, Caribbean, South America)? How do these numbers compare among regions? Do these numbers correspond to the currently recognized number of species? What does this mean in the context of the genetic species concept and species-level variation in *Myotis*? One species in particular that we focus on is *Myotis nigricans*, a species that is hypothesized to have an expansive distribution throughout the Neotropics [1], [13]. Hence, is this species a monophyletic unit across its geographic range? Are genetic distance values of purported *M. nigricans* representative of intraspecific variation?

To answer these questions, we used the genetic species concept to test general hypotheses about intraspecific variability [17]–[21]). In addition to *M. nigricans*, we examined an expanded sample of several *Myotis* species (*M. albescens*, *M. keaysi*, *M. riparius*) from different geographic locations in South America to provide insight into the number of potential species-level lineages present on this continent. Finally, we explored sequence divergence across our sample and compared our results with previously published research on New World *Myotis*.

## Methods

**Figure 1:**
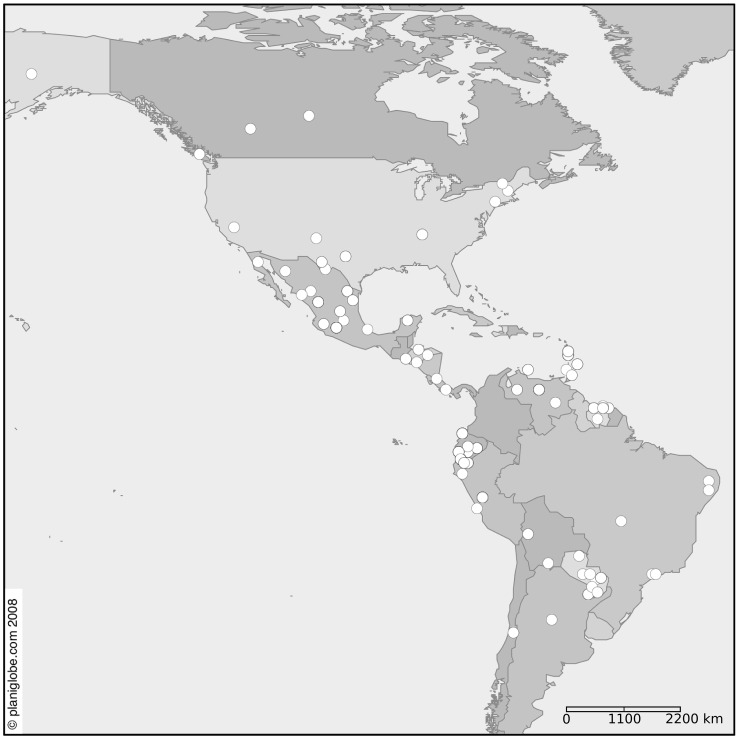
Distribution of collecting localities for specimens used in this study. Numbers of specimens from each general region are as follows: South America (135), North America (51), Caribbean (17), and Central America (8). Four specimens were Old World representatives and are not shown. Map generated using the planiglobe ® (http://www.planiglobe.com/omc_set.html) digital vector map tool.

For this study, we utilized the museum collection of tissue vouchers from the Genetic Resources Collection of the Natural Science Research Laboratory (NSRL) of Texas Tech University. All tissues loaned for this study, as well as associated DNA, are archived at the NSRL. Loans from the Genetics Resources Collection require approval of the executive director of the Museum of Texas Tech University and loans of all such tissues were approved. Specimens examined consisted of *Myotis* collected from throughout North, Central, South America, and the Caribbean (Figure 1). Associated museum voucher specimens are held in public and private museums, and were collected over a span of several decades in collaboration with multiple individuals and institutions. Geographic locality, museum voucher number, tissue number, and GenBank accession numbers for all specimens examined are listed in Tables S1 and S2.

### Molecular Methods

Genomic DNA was extracted from liver, muscle, or wing punches following the methods of Longmire et al. [22], or by using the DNeasy Blood and Tissue Kit (Qiagen Inc., Chatsworth, California). External and internal primers used to amplify and sequence *Myotis* specimens, as well as PCR methods, are reported in Larsen et al. [16]. PCR products were purified using a QIAquick PCR Purification Kit (Qiagen Inc., Chatsworth, California) or ExoSAP-IT (Affymetrix, Inc., Santa Clara, California).

DNA sequencing of cytochrome-*b* was performed using ABI Big Dye chemistry chain terminators version 3.1 and fragments were electrophoresed on an ABI 3100-*Avant* Genetic Analyzer (PE Applied Biosystems, Foster City, California). Sequences were verified and assembled using Sequencher 4.10.1 (Gene Codes Corporation, Ann Arbor, Michigan). Multiple sequence alignments were performed manually and verified in MacClade version 4.08 OS X [23].

### Phylogenetic Analyses

Fifty-seven sequences were gathered from previously published GenBank sequences used in subsequent phylogenetic analyses [2], [3], [16]. Reference sequences for currently recognized species lineages consisted of cytochrome-*b* sequences from Stadelmann et al. [3] and Ruedi and Mayer [2]. One hundred and fifty-eight sequences were generated for this study, with 86 being used in final analyses. Following Stadelmann et al. [3], some of the 158 sequences we excluded as sequence divergences were below 1% (based on Kimura 2-parameter pairwise comparisons [24]). Exceptions were made when individuals were collected from distinct collecting localities, as they provided insight into intraspecific variation. All sequences generated for this study were submitted to GenBank (Accession numbers JX130435–130592 in Tables S1 and S2).

Phylogenetic analyses were performed using MrBayes version 3.1.2 [25], MEGA version 5.0 software [26], [27], and PAUP* version 4.0b10 [28]. Maximum likelihood, maximum-parsimony (unweighted), and Bayesian analyses were used to infer phylogenies. Bootstrap support values (≥75%) and Bayesian posterior probabilities (≥0.95) were used to measure statistical support. Genetic distance values for cytochrome-*b* were generated in MEGA using the Kimura 2-parameter, which allowed for comparisons with previous molecular studies of *Myotis* [2], [3], [20], [29], [30] and other mammalian taxa [17]. To best assess the genetic diversity within the wide-ranging Neotropical *Myotis*, *M. nigricans*, we used a genetic representative available from GenBank (*M. nigricans* from Ruedi and Mayer [2]; AF376864) to compare *M. nigricans*-types throughout the Neotropics [2], [3], [31], [32].

Maximum-parsimony analysis was performed using heuristic searches, 25 replicates of the random taxon addition option, each with random starting trees, and tree-bisection-reconnection branch swapping. For bootstrap support values, 1,000 replicates were conducted using the heuristic search criterion. Nucleotide substitution models were analyzed in MEGA to determine the appropriate model of evolution for the cytochrome-*b* gene. Based on the Bayesian Information Criterion (BIC), the GTR+G+I model was chosen. The GTR model was used in MEGA to run 1,000 bootstrap iterations and obtain bootstrap support values. Bayesian analyses of sequence data were performed to obtain posterior probabilities and consisted of one run with four Markov chain Monte Carlo chains (one heated and three cold) run for 2 million generations. Trees were sampled every 100 generations with a burn-in value of 1,000 trees. We explored the level of saturation in the cytochrome-*b* dataset using Xia’s method [33], [34] as implemented in DAMBE version 5.2 [35] and determined the consistency and retention indices in MEGA to test for homoplasy.

### Descriptive Analyses

Pairwise distance values were calculated in MEGA and a histogram of the total number of pairwise distances versus Kimura 2-parameter distances was created. Tables were tabulated after distance analyses and visual inspection of the Neighbor-joining phylogram. Voucher information and regional keys were used to obtain baseline morphological identifications. We referred to previous studies of species-level genetic variation in mammals (including *Myotis*) [3], [17]–[20] and subsequently used average interspecific sequence divergence values (e.g., 2.0% and 5.0%) as a general starting point for determining the number of putative species-level lineages present in our sample. The use of the genetic species concept and these values are not meant to be a strict delimitation, but are a guide to help estimate the number of possible genetic lineages (i.e., species) in our sample.

## Results

### Phylogenetic Analyses

**Figure 2:**
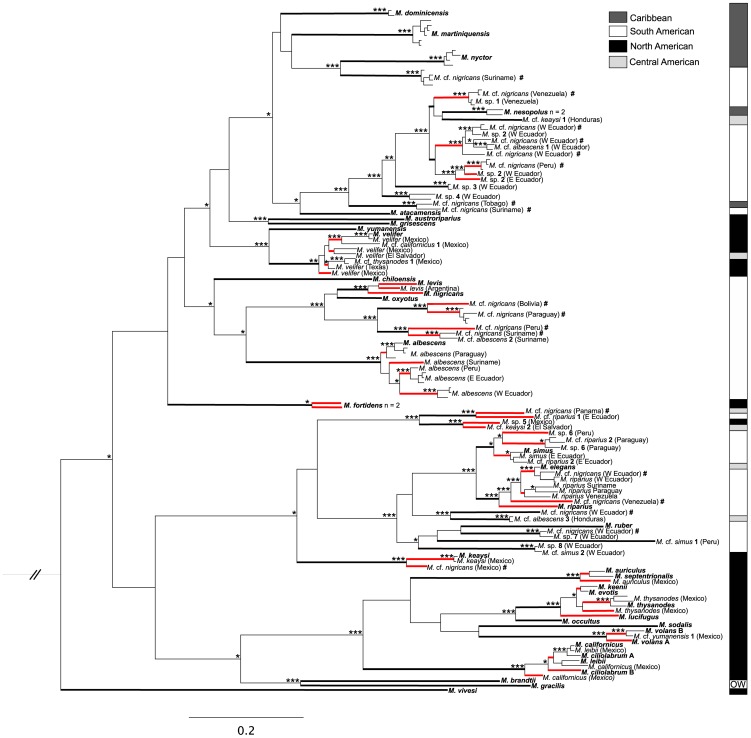
Bayesian phylogram of cytochrome-*b* sequence data (n = 140). Species names are based on museum and field records. Bolded species names are currently recognized by Simmons [13] and serve as the species representative (from GenBank [2], [3], [16] or from specimens sequenced herein). Black bolded branches are lineages that are ≥5.0% divergent and red bolded branches are lineages ≥2.0% divergent. Bolded numbers within species names indicate specimens that were originally identified as the named species, but were found to be independent lineages that are ≥5.0% divergent. The pound sign (**#**) indicates all specimens originally identified as *M. nigricans*. OW = Old World lineages of *M. brandtii* and *M. gracilis*. *Kerivoula papillosa* and *M. latirostris* are outgroup taxa but are not shown in the phylogram. Support for nodes are presented as Bayesian posterior probabilities ≥0.95 (*), maximum likelihood bootstrap pseudo-replicate values ≥75% (**), or support in both analyses (***). Additional specimen information is in Table S1.

**Figure 3:**
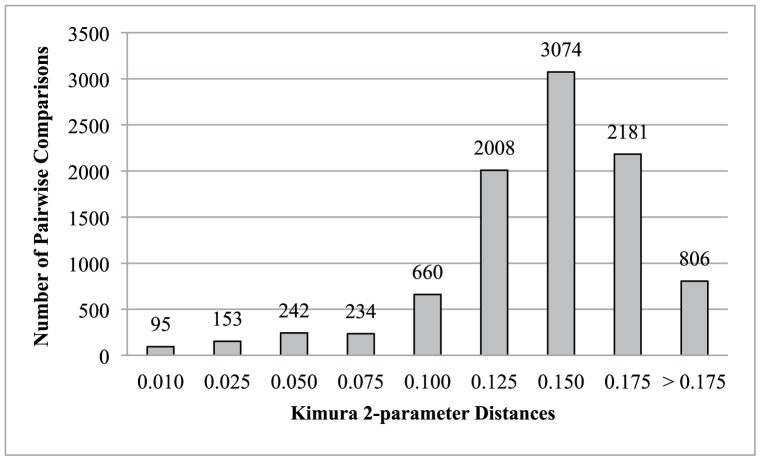
Kimura 2-parameter pairwise distances based on cytochrome-*b* sequence data. Y-axis indicates the number of total pairwise comparisons of sequences and the X-axis indicates the Kimura 2-paramter distance values among ingroup taxa. Note the large portion of pairwise values over 10% (average divergence of 13.14%).

Alignment of all sequences was unequivocal and without internal stop codons. Of the 215 sequences initially scanned, 170 consisted of the entire cytochrome-*b* gene (1,140 base pairs [bps]), whereas 45 sequences contained between 500 and 1,120 bps. The final dataset consisted of 140 sequences with unique haplotypes (124 complete and 16 partial sequences of the cytochrome-*b* gene). Excluding outgroups (*Kerivoula papillosa* and *Myotis latirostris*), 456 characters in cytochrome-*b* were parsimony-informative with 89 at position one, 22 at position two, and 345 at position three. Parsimony analysis generated 23 most parsimonious trees of 3,488 steps (retention index = 0.78, consistency index = 0.19). The consistency index indicated a high level of homoplasy, but analyses of saturation indicated that the index of substitution saturation was significantly less than the critical value of index substitution saturation (Xia’s test as implemented in DAMBE; *P*<0.01). Topology of the strict consensus of the 23 equally parsimonious trees was similar to trees generated in all analyses. Maximum-likelihood analyses resulted in a single optimal tree (−*ln*L = 16,687.563; Figure 2) based on 287 parameters, with a proportion of invariable sites of 53.5% and a gamma distribution parameter of 0.524. Cytochrome-*b* genetic distances were relatively high (see Figure 3 - the majority were ≥10.0%). The average Kimura 2-parameter genetic distance value among all in-group specimens was 13.14% (standard error 1.26; Table 1). The range of average distance values by region was 8.13% (Caribbean) to 13.73% (North America; Table 1).

### Descriptive Analyses

**Figure 4:**
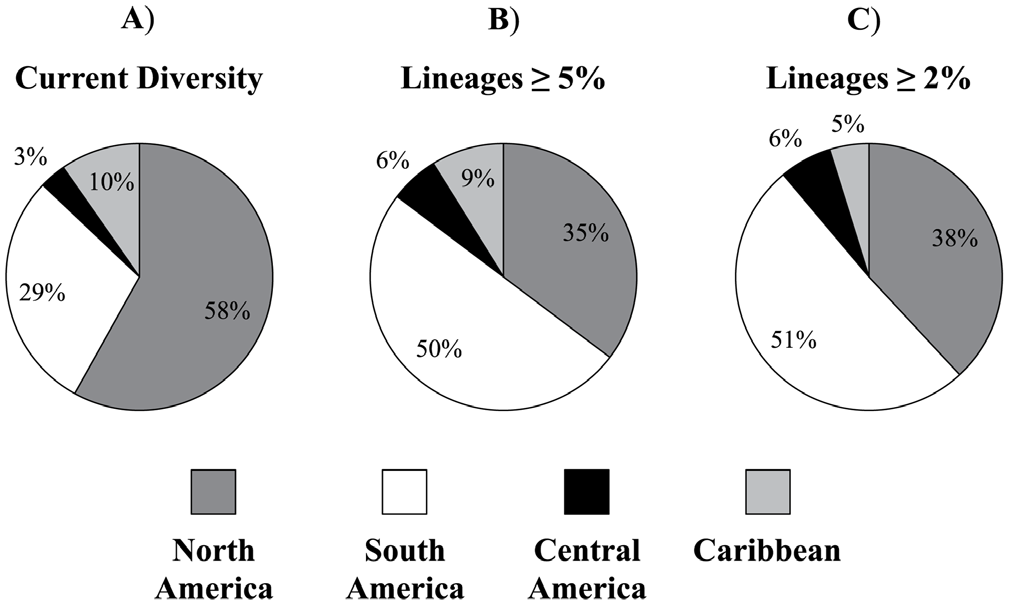
Diversity estimates based on currently recognized species and genetic lineages presented herein. The number of lineages restricted to each region (distributions based on [12]–[16]) is shown as a percentage of the total. **A**) percentage of diversity in each region based on currently recognized diversity, **B**) percentage of diversity in each region based on a species divergence level set at ≥5.0% using cytochrome-*b* DNA sequence data, **C**) percentage of diversity in each region based on a species divergence level set at ≥2.0%. Note the proportion of lineages from each region changes from the current levels, when compared to the lineages at 5.0% and 2.0%.

The approximate number of *Myotis* species endemic (distribution is restricted) to the following regions based on the current literature are: North America = 18, Central America = 1, Caribbean = 3, and South America = 9 (Table 1, percentages shown in Figure 4A; [12]–[16]). Eleven additional species have geographic ranges that overlap in two or more regions and are not included in the percentage of the total. The percentage of species-level lineages in South America increases when comparing those identified from previous publications (Figure 4A) to those based on the data reported herein (at two levels of genetic distance values: 5.0% [34 total lineages] and 2.0% [63 total lineages]; Figures 4B and C, respectively). The number of lineages ≥5.0% in Kimura 2-parameter genetic distance by region (also excludes taxa with ranges overlapping in more than one region) were as follows: North America = 12, Central America = 2, Caribbean = 3, and South America = 17 (percentages shown in Figure 4B). For lineages ≥2.0%, these values are: North America = 24, Central America = 4, Caribbean = 3, and South America = 32 (percentages shown in Figure 4C).

Within the most widely distributed Neotropical species (*M. albescens*, *M. keaysi*, *M. nigricans*, and *M. riparius*), several lineages ≥5.0% (within each of these species complexes) were recovered (Table 2). For example, of 17 specimens identified as *M. albescens* from Central and South America, 4 lineages were ≥5.0% divergent from each other (if the genetic distance separating lineages was set at ≥2.0%, 7 lineages were recovered). Of 16 *M. nigricans* specimens (collected from North, Central, and South America, and the Caribbean), 8 lineages were ≥5.0% divergent from each other and 11 lineages were ≥2.0% divergent (Table 2). In *M. keaysi*, three lineages were ≥5.0% divergent from each other (these same three were divergent at ≥2.0%), and in *M. riparius*, three lineages were ≥5.0% divergent from each other (6 were at ≥2.0%).

**Table 2:** Species lineage information based on cytochrome-*b* analyses.

Name	Recognized Subspecies	Distribution	n	General Colleting Locality	≥2%	≥5%
*M. albescens*		southern MX, CA to SA	17	Bolivia*, Ecuador, Honduras, Paraguay, Peru, Suriname	7	4
*M. auriculus*	2	south-western US, MX to northern CA	2	Mexico*	2	1
*M. californicus*	4	western US and MX	4	Mexico*	3	2
*M. dominicensis*		Dominica, Guadeloupe	2	Dominica*	1	1
*M. fortidens*	2	southern MX to northern CA	2	Mexico	2	1
*M. keaysi*	2	north-eastern MX, CA, to north and central SA, Trinidad	4	El Salvador, Honduras, Mexico*	3	3
*M. leibii*		eastern NA	2	Mexico, New York*	1	1
*M. levis*	2	central SA	2	Argentina, Brazil*	2	1
*M. martiniquensis*		Martinique, Barbados	6	Martinique*	1	1
*M. nesopolus*	2	Bonaire, Curacao, Venezuela	2	Bonaire*	1	1
*M. nigricans*	3	central MX, CA to SA, Trinidad and Tobago, St.Martin, Montserrat, Grenada	29	Bolivia, Brazil*, Ecuador, Mexico, Panama, Paraguay,Peru, Suriname, Tobago, Venezuela	16	12
*M. nyctor*		Barbados, Grenada	4	Barbados*, Grenada	1	1
*M. riparius*		CA to north and central SA, Trinidad and Tobago	10	Brazil*, Ecuador, Paraguay, Suriname, Venezuela	6	3
*M. simus*		Amazon basin of central SA and south-central SA	4	Brazil*, Ecuador, Peru	3	3
*M.* sp.			14	Ecuador, Mexico, Paraguay,Peru, Venezuela	11	8
*M. thysanodes*	4	NA	6	Mexico, Texas*	3	2
*M. velifer*	5	south-western and south-central US to central CA	6	El Salvador, Mexico*, Texas	4	1
*M. yumanensis*	6	western NA	2	California*, Mexico	2	2

Note the number of lineages within each species with ≥5.0% and 2.0% divergence values. Number of currently recognized subspecies are based on Simmons [13] and distributions are based on Simmons [13] and Wilson [14]. n = number of individuals sequenced from each species. Asterisks indicate the general collecting locality of GenBank specimens. Abbreviations: MX = Mexico, US = United States.

Within a single monophyletic clade of *M. albescens* collected from Bolivia, Ecuador, Paraguay, Peru, and Suriname (Figure 2), <5.0% genetic divergence was present among all its members. Less than 5.0% divergence was also present in two separate clades that have currently recognized species from Central and South America, but these clades are paraphyletic as they contain more than one recognized species (see bolded: *M. levis* and *M. nigricans* near middle of phylogram; *M. elegans* and *M. riparius* near lower third of phylogram; Figure 2). Paraphyletic assemblages with <5% divergence are also present in 3 separate clades containing multiple recognized species from North America (see bolded: *M. auriculus* and *M. septentrionalis*; *M. keenii*, *M. evotis*, *M. thysanodes*, and *M. lucifugus*; and *M. californicus*, *M. ciliolabrum*, and *M. leibii*; all near lowest branches of the phylogram in Figure 2).

## Discussion

Our data provide further evidence that the number of Neotropical (specifically South American) species of *Myotis* is likely underestimated. It is important to state again that not all divergence values ≥2.0% or 5.0% as detected in a cytochrome-*b* dataset represent unrecognized species. However, the lineages recovered in our data do indicate maternally independent evolutionary trajectories with sufficient genetic distances to warrant further study. To this end, if highly divergent mitochondrial DNA indicates unrecognized genetic-based species [18]–[20], [31], [36], minimally there are 37 lineages with ≥5.0% divergence in our sample and up to 67 lineages with ≥2.0% divergence. Many of these lineages likely represent unrecognized taxa (especially those with ≥11.0% divergence [17]), however, a multi-faceted research approach (i.e., suites of molecular, morphological, and ecological data) will be needed to fully resolve the relationships identified using mitochondrial DNA sequences.

Although previous studies suggest that diversity at the species level in South American *Myotis* has been less than half that of North American *Myotis* [1], [4], [13], [14]), there is evidence that this disparity is directly attributable to a lack of research and collection of South American representatives [1], [3], [16]. The results herein indicate that there are at least as many lineages with ≥5.0% divergence in the cytochrome-*b* gene in South America as there are in North America, suggesting more unrecognized species are present in South America (Figures 2 and 4, Tables 1 and 2). Notably, four traditionally recognized and widely distributed species of Neotropical *Myotis* (*M. albescens*, *M. keaysi*, *M. nigricans*, and *M. riparius*) are paraphyletic and may be comprised of multiple independent evolutionary lineages with ≥5.0% divergence values (Figure 2, Tables 1 and 2).

Genetic distance values as low as 2.0% separate currently recognized species of *Myotis*. If this distance value accurately delineates two species, then a value of 5.0% will be an even more conservative estimate (in mitochondrial genes) for exploring species-level variation [2], [19], [20], [36] in New World *Myotis*. We discuss the diversity present in our dataset from South America, and further investigate *Myotis nigricans*, the most widely distributed and common Neotropical species within the genus. Additionally, we discuss the implications to broad scale diversity patterns based on our sample.

### Species Richness in South America

Chiropteran diversity is high in the Neotropics [13], [20], [37] and our study indicates South American *Myotis* are no exception to this pattern, as the genus likely contains more species than previously reported. For example, of the most widely distributed species from South America (*M. albescens*, *M. nigricans*, and *M. riparius*), each contains at least 3 lineages separated by genetic distances greater than 5.0% (Table 2). Of these, *M. nigricans* does not show monophyly among all its members, but contains 12 species-level lineages with greater than 5.0% divergence (Figure 2, Table 2). On the other hand, two distinct and independently monophyletic clades represent *M. albescens* (from Bolivia, Ecuador, Paraguay, Peru, and Suriname [Figure 2]) and *M. velifer* (from El Salvador, Mexico, and Texas [Figure 2]). This observation also fits within the traditionally defined geographic boundaries for both *M. albescens* and *M. velifer* [13], [14].

In general, most traditionally recognized South American lineages are more than 5.0% divergent from each other, whereas traditionally recognized North American species display relatively low levels of interspecific sequence divergence (typically <2.0%; see Figure 2). This observation is interesting as previous hypotheses based on morphology and molecular data indicate that North American lineages have had more time to diversify and speciate than have lineages in South America [1], [3], [37]. If this hypothesis is accurate, then the observation of a greater number of distinct genetic lineages (i.e., >5%) in South America with respect to North America may indicate either an incomplete sampling effort or a greater number of extinction events within the North American *Myotis* fauna. Alternatively, the South American fauna may have diversified more rapidly than North American *Myotis* lineages due to a greater diversity of habitats that support specialization to available niches.

Overall, genetic distance values for our entire sample (excluding outgroup members) average greater than 10.0% (>90% of all pairwise comparisons), with most residing between 15.0% and 17.5% (Figure 3). These pairwise comparisons are at or above accepted species-level divergence values [2], [18]. Our phylogeny contains 34 traditionally recognized species (32 New World and 2 Old World), as well as the recently elevated *Myotis nyctor* [16]. Based on our sample and the GenBank specimens available, the number of taxa recognized in North America with cytochrome-*b* distances ≥2.0% divergence is 25, with 34 recognized at this level in South America. If divergence criteria were increased to ≥5.0%, 13 taxa would be recognized in North America, with 18 recognized in South America. In both examples, the South American *Myotis* fauna increases, whereas North America contains less than the currently recognized number of species. The disparity between recognized and genetically distinct species in our sample requires further examination of South American *Myotis* both morphologically and molecularly, especially those species that are thought to be common and widely distributed (e.g., *Myotis nigricans*).

### The Myth of Myotis Nigricans

The intraspecific variation of *M. nigricans* is currently partitioned into three subspecies (*nigricans*, *extremus*, *osculatii*), with approximately 20 synonyms listed for the species [5], [13], [14]. *Myotis nigricans*, as currently defined, is distributed throughout Mexico, Central America, in all South American countries except Chile and Uruguay, and possibly the southern Caribbean [13], [14], [16]. In terms of square kilometers, the geographic range of *M. nigricans* is the largest of any species range for New World *Myotis*. However, the expansive geographic distribution of *M. nigricans* is likely a failure to distinguish unrecognized species. A case in point is LaVal’s work [1: p. 6], where he noted “Any specimen that does not seem to fit the diagnosis of another species is probably *nigricans*.” If LaVal truly meant this, then we would expect that genetic data would be compatible with a hypothesis that *Myotis nigricans* from throughout its distribution would: 1) constitute a monophyletic unit, and 2) have genetic distance values representative of intraspecific variation (i.e., similar to those found in *Myotis* and other bat species with a comparably wide geographic distribution; e.g., *Artibeus lituratus*, *Carollia perspicillata* [20], [38]).

Our genetic analysis of the cytochrome-*b* gene from museum holdings of several South American *Myotis nigricans* revealed multiple paraphyletic assemblages with large intraspecific genetic distances that are likely an artifact of such taxonomic treatments (Figure 2 and Table 2). These DNA sequence data indicate that *M. nigricans* likely has a more restricted distribution than previously thought and may only be found in a small region of South America (i.e., south-eastern Brazil). If this hypothesis is accurate, then the identification of all little brown *Myotis* as ‘*M. nigricans*’ (although convenient) does not accurately reflect the diversity of small, brown, and morphologically similar Neotropical *Myotis*. Knowing this, it would be valuable to examine the presence/absence of morphological variation of representative specimens of the lineages referred to as *M. nigricans* presented herein. Additional combined studies of morphology and genetics would be useful in this respect, as recent morphological studies have suggested intraspecific cohesion patterns in skull shape across *M. nigricans* and other South American species of *Myotis* [12], [39], [40].

### Summary and Future Implications

Results of our analyses of cytochrome-*b* sequence data from 32 recognized species of New World *Myotis* indicate the presence of at least 15 lineages that likely represent unrecognized or cryptic species. The need for more extensive geographic sampling in addition to more thorough morphological, genetic, and genomic studies of South American and Neotropical *Myotis* is evident [3], [10], [11], [20]. An incomplete knowledge of the diversity and species richness of *Myotis* from the Neotropics compounds the difficulty in resolving their relationships, biogeography, evolutionary history, and conservation status. Our study provides a platform to address these issues, by supplying a large amount of mitochondrial sequence data with respect to South American *Myotis*. These data can be used not only for further taxonomic study, but also in combination with standard morphological examinations and geometric morphometrics [12], behavioral considerations (e.g., echolocation frequency; [41]), genomic approaches [42], [43], and new statistical methods [44], [45] to help provide more resolution for cryptic lineages and support for relationships among *Myotis* as well as other New World vespertilionids. We hypothesize that the diversity within *Myotis* worldwide is underestimated in a similar fashion as we have found in this paper. Therefore, a more accurate description of this diversity will help us better understand the adaptive nature of *Myotis* species and the dynamic forces impacting island and continental fauna, in addition to ultimately helping protect and conserve *Myotis* species diversity in less well-studied regions.

## Supporting Information

Table S1 Specimens examined.
(DOCX)

Table S2 Additional specimens sequenced but not used in final genetic analyses.
(DOCX)
