# Supplementary material for: Genetic Diversity of Neotropical Myotis (Chiroptera: Vespertilionidae) with an Emphasis on South American Species
Source: PLoS One. 2012 Oct 3;7(10):e46578. doi: 10.1371/journal.pone.0046578 (PMC3463536; doi:10.1371/journal.pone.0046578)
Supplement: Table S1 — Specimens examined. (DOCX) [file pone.0046578.s001.docx]

**Table S1. Specimens used in cytochrome-*b* analyses**. Abbreviations and acronyms for institutions or individual collections are as follows: François M. Catzeflis, Universite of Montpellier: Laboratoire de Paléontologie, France (Catzeflis-Montpellier-V-); Instituto Politecnico Nacional in Mexico (CDR); Carnegie Museum of Natural History (CM); Field Museum of Natural History (FMNH); Jean-François Maillard (JFM DIREN); Kunico Kawai (KK); Museum National Histoire Naturelle, Paris, France (MNHN); Manuel Ruedi (MR); Museum of Vertebrate Zoology (MVZ); National Museum Prague (NMP); Museo de Zoologia, Escuela de Biologia, Pontificia Universidad Catolica del Ecuador (QCAZ); Royal Ontario Museum (ROM); T. H. Kunz (THK); Museum of Texas Tech University (TTU = voucher specimen, TK = tissue); University of Alaska Museum (UAM); Universidad Autonoma Metropolitana-Iztapalapa (UAMI); and University of Nebraska State Museum (UNSM ZM). N/A = Not Available. Previously published GenBank specimens appear in [2,3,16,32,33]. Bolded specimens and numbered lineages correspond to individuals in Figure 2.

| **Name, Clade No., Locality** | **Specific Locality** | **Voucher No.** | **Tissue No.** | **Accession No.** |
| --- | --- | --- | --- | --- |
| ***K. papillosa*** | Dong Amphan, S. Laos | ROM 110520 | – | AJ841969 |
| ***M. albescens*** | Tarija, Bolivia | FMNH 162543 | – | AF376839 |
| *M.* cf. *albescens* **1** W Ecuador | Esmeraldas Province, Ecuador | TTU 85348 | TK 104576 | JX130466 |
| *M.* cf. *albescens* **1** W Ecuador | Esmeraldas Province, Ecuador | TTU 85410 | TK 104638 | JX130467 |
| *M.* cf. *albescens* **2** Suriname | Marowijne, Suriname | CM 77691 | TK 17932 | JX130497 |
| *M.* cf. *albescens* **3** Honduras | Atlántida, Honduras | TTU 84138 | TK 101479 | JX130480 |
| *M.* cf. *albescens* **3** Honduras | Atlántida, Honduras | TTU 84380 | TK 101723 | JX130479 |
| *M. albescens* E Ecuador | Pastaza District, Ecuador | TTU 85088 | TK 104316 | JX130463 |
| *M. albescens* E Ecuador | Pastaza District, Ecuador | TTU 85089 | TK 104317 | JX130464 |
| *M. albescens* E Ecuador | Pastaza District, Ecuador | TTU 85094 | TK 104322 | JX130465 |
| *M. albescens* Paraguay | Ñeembucú, Paraguay | TTU 99818 | TK 61766 | JX130504 |
| *M. albescens* Paraguay | Boquerón, Paraguay | TTU 99124 | TK 62827 | JX130503 |
| *M. albescens* Paraguay | Ñeembucú, Paraguay | TTU 99801 | TK 64429 | JX130502 |
| *M. albescens* Peru | Huánuco Dept., Peru | TTU 46343 | TK 22835 | JX130445 |
| *M. albescens* Suriname | Nickerie, Suriname | CM 63920 | TK 10319 | JX130444 |
| *M. albescens* W Ecuador | Guayas, Ecuador | TTU 103744 | TK 134650 | JX130501 |
| *M. albescens* W Ecuador | El Oro, Ecuador | TTU 102348 | TK 135124 | JX130500 |
| *M. albescens* W Ecuador | El Oro, Ecuador | TTU 102363 | TK 135139 | JX130472 |
| ***M. atacamensis*** | Olmos, Peru | MVZ 168933 | – | AM261882 |
| ***M. auriculus*** | Guanaceví, Mexico | CDR 3288 | – | AM261884 |
| *M. auriculus* Mexico | Nuevo Leon, Mexico | TTU 104855 | TK 150002 | JX130482 |
| ***M. austroriparius*** | Tennessee, USA | THK 002 | – | AM261885 |
| ***M. brandtii*** | N.W. Russia | NMP PB916 | – | AM261886 |
| ***M. californicus*** | Durango, Mexico | CDR 3276 | – | AM261887 |
| *M.* cf*. californicus* **1** Mexico | Sinaloa, Mexico | TTU 35585 | TK 4703 | JX130441 |
| *M.* cf*. californicus* **2** Mexico | Nuevo Leon, Mexico | TTU 57051 | TK 32531 | JX130443 |
| *M.* *californicus* Mexico | Querétaro, Mexico | TTU 35586 | TK 4834 | JX130442 |
| ***M. chiloensis*** | Santiago, Chile | THK I | – | AM261888 |
| ***M. ciliolabrum* A** | Canada | – | – | AM261889 |
| ***M. ciliolabrum* B** | Durango, Mexico | CDR 3172 | – | AM261890 |
| *M. dominicensis* | St. Joseph Parish, Dominica | TTU 31519 | TK 15577 | JN020554 |
| ***M. dominicensis*** | St. Joseph Parish, Dominica | – | TK 15613 | AF376848 |
| ***M. elegans*** | La Selva, Costa Rica | MR-3011 | – | AM261891 |
| ***M. evotis*** | Alberta, Canada | – | – | AJ841949 |
| *M. fortidens* | Michoacán, Mexico | UAMI (R. Lopez-Wilchis) | TK 43134 | JX130437 |
| *M. fortidens* | Michoacán, Mexico | UAMI (R. Lopez-Wilchis) | TK 45364 | JX130439 |
| ***M. gracilis*** | Hokkaido, Japan | KK 0005 | ­– | AB106609 |
| **Name Clade No. Locality** | **Specific Locality** | **Voucher No.** | **Tissue No.** | **Accession No.** |
| ***M. grisescens*** | Tennessee, USA | THK 11500 | – | AM261892 |
| ***M. keaysi*** | Yucatan, Mexico | TK 13532 | – | AF376852 |
| *M.* cf*. keaysi* **1** Honduras | Valle, Honduras | TTU 61228 | TK 40198 | JX130493 |
| *M.* cf*. keaysi* **2** El Salvador | Santa Ana, El Salvador | TTU 60981 | TK 34869 | JX130519 |
| *M.* *keaysi* Mexico | Yucatan, Mexico | TTU 47514 | TK 13526 | JX130449 |
| ***M. keenii*** | Vancouver Island, USA | THK 98-44 | – | AM262329 |
| ***M. latirostris*** | Moi-Li Co, Taiwan | MR-608 | – | AM262330 |
| ***M. leibii*** | West Point, NY, USA | THK 2 | – | AM262331 |
| *M. leibii* Mexico | Durango, Mexico | TTU 75560 | TK 48574 | JX130440 |
| ***M. levis*** | Sao Paulo, Brazil | FMNH 141600 | – | AF376853 |
| *M. levis* Argentina | Córdoba, Argentina | TTU 66489 | TK 49150 | JX130475 |
| ***M. lucifugus*** | Alaska, USA | UAM 22927 | – | AF376854 |
| ***M. martiniquensis*** | Martinique, France DOM | JFM DIREN 2 | – | AM262332 |
| *M. martiniquensis* | Le Morne-Rouge, Martinique | MNHN: 2005-896 | – | JN020558 |
| *M. martiniquensis* | GranďRivière, Martinique | Catzeflis-Montpellier-V-2352 | – | JN020559 |
| *M. martiniquensis* | GranďRivière, Martinique | MNHN: 2008-974 | – | JN020560 |
| *M. martiniquensis* | GranďRivière, Martinique | Catzeflis-Montpellier-V-2354 | – | JN020561 |
| *M. martiniquensis* | Le Morne-Rouge, Martinique | MNHN: 2005-895 | – | JN020557 |
| *M. nesopolus* | Bonaire, Dutch Caribbean | – | TK 128151 | JN020575 |
| *M. nesopolus* | Bonaire, Dutch Caribbean | – | TK 128152 | JN020576 |
| ***M. nigricans*** | Paraiba, Brazil | MVZ AD50 | – | AF376864 |
| *M.* cf. *nigricans* Bolivia | La Paz, Bolivia | TTU 34952 | TK 14529 | JX130450 |
| *M.* cf. *nigricans* Mexico | Vera Cruz, Mexico | CM 55764 | TK 13147 | JX130489 |
| *M.* cf. *nigricans* Panama | Chiriquí, Panama | TTU 39146 | TK 22503 | JX130435 |
| *M.* cf. *nigricans* Paraguay | Ñeembucú, Paraguay | TTU 99802 | TK 61647 | JX130499 |
| *M.* cf. *nigricans* Paraguay | Alto, Paraguay | TTU 99046 | TK 63261 | JX130498 |
| *M.* cf. *nigricans* Paraguay | Presidente Hayes, Paraguay | TTU 99743 | TK 64475 | JX130496 |
| *M.* cf. *nigricans* Paraguay | Dept. San Pedro, Paraguay | TTU 95992 | TK 99628 | JX130455 |
| *M.* cf. *nigricans* Peru | Huánuco Dept., Peru | TTU 46347 | TK 22702 | JX130452 |
| *M.* cf. *nigricans* Peru | Huánuco Dept., Peru | CM 98859 | TK 22704 | JX130453 |
| *M.* cf. *nigricans* Peru | Huánuco Dept., Peru | CM 98858 | TK 22840 | JX130454 |
| *M.* cf. *nigricans* Peru | Huánuco Dept., Peru | CM 98860 | TK 22952 | JX130484 |
| *M.* cf. *nigricans* Suriname | Nickerie, Suriname | CM 63933 | TK 10123 | JN020570 |
| *M.* cf. *nigricans* Suriname | Para, Suriname | CM 69053 | TK 17272 | JN020571 |
| *M.* cf. *nigricans* Suriname | Marowijne, Suriname | CM 77692 | TK 17556 | JX130476 |
| *M.* cf. *nigricans* Suriname | Paramaribo, Suriname | CM 77705 | TK 17783 | JX130505 |
| *M.* cf. *nigricans* Suriname | Para, Suriname | CM 77699 | TK 21050 | JN020572 |
| *M.* cf. *nigricans* Suriname | Sipaliwini, Suriname | TTU 109227 | TK 151465 | JX130520 |
| *M.* cf. *nigricans* Tobago | St. George Parish, Tobago | UNSM ZM-29470 | TK 157625 | JN020573 |
| *M.* cf. *nigricans* Venezuela | Guárico, Venezuela | TTU 48161 | TK 15074 | JN020569 |
| *M.* cf. *nigricans* Venezuela | Guárico, Venezuela | TTU 48166 | TK 15256 | JX130494 |
| *M.* cf. *nigricans* Venezuela | Barinas, Venezuela | CM 78651 | TK 19442 | JX130490 |
| *M.* cf. *nigricans* W Ecuador | Guayas, Ecuador | TTU 103759 | TK 134759 | JX130470 |
| *M.* cf. *nigricans* W Ecuador | Guayas, Ecuador | TTU 103483 | TK 135006 | JX130457 |
| *M.* cf. *nigricans* W Ecuador | Loja, Ecuador | TTU 103805 | TK 135301 | JX130447 |
| *M.* cf. *nigricans* W Ecuador | El Oro, Ecuador | TTU 102424 | TK 135364 | JX130446 |
| *M.* cf. *nigricans* W Ecuador | Esmeraldas Province, Ecuador | TTU 102833 | TK 135514 | JX130448 |
| *M.* cf. *nigricans* W Ecuador | Esmeraldas Province, Ecuador | TTU 102883 | TK 135564 | JX130492 |
| *M.* cf. *nigricans* W Ecuador | Chimborazo, Ecuador | QCAZ 11383 | TK 161650 | JX130517 |
| ***M. nyctor*** | St. David Parish, Grenada | CM 83427 | TK 125846 | JN020562 |
| *M. nyctor* | St. Thomas Parish, Barbados | TTU 109225 | TK 151413 | JN020563 |
| *M. nyctor* | St. Joseph Parish, Barbados | TTU 109229 | TK 161049 | JN020565 |
| **Name Clade No. Locality** | **Specific Locality** | **Voucher No.** | **Tissue No.** | **Accession No.** |
| *M. nyctor* | St. Thomas Parish, Barbados | TTU 109230 | TK 161125 | JN020567 |
| ***M. occultus*** | New Mexico, USA | THK 25 | – | AM262334 |
| ***M. oxyotus*** | Lima, Peru | FMNH 129208 | – | AF376865 |
| ***M. riparius*** | Pernambuco, Brazil | MVZ AD119* | – | AF376866 |
| *M.* cf. *riparius* **1** E Ecuador | Tungurahua Province, Ecuador | TTU 85060 | TK 104288 | JX130514 |
| *M.* cf*. riparius* **2** Paraguay | Dept. Canindeyú, Paraguay | TTU 94912 | TK 56784 | JX130486 |
| *M.* cf*. riparius* **2** Paraguay | Paraguarí, Paraguay | TTU 99645 | TK 63827 | JX130485 |
| *M.* cf. *riparius* **2** E Ecuador | Pastaza District, Ecuador | TTU 84870 | TK 104098 | JX130513 |
| *M. riparius* Paraguay | Dept. Canindeyú, Paraguay | TTU 99378 | TK 63402 | JX130491 |
| *M. riparius* Suriname | Para, Suriname | CM 68443 | TK 17370 | JX130473 |
| *M. riparius* Venezuela | Bolivar, Venezuela | CM 78659 | TK 19215 | JX130474 |
| *M. riparius* W Ecuador | Esmeraldas Province, Ecuador | TTU 85344 | TK 104572 | JX130469 |
| *M. riparius* W Ecuador | Esmeraldas Province, Ecuador | TTU 85345 | TK 104573 | JX130515 |
| ***M. ruber*** | Salesópolis, Brazil | MVZ AD472* | – | AF376867 |
| ***M. septentrionalis*** | New Hampshire, USA | THK 1 | – | AM262335 |
| ***M. simus*** | Brazil | THK Mys-ET3 | – | AM262336 |
| *M.* cf. *simus* **1** Peru | Huánuco Dept., Peru | TTU 46348 | TK 22688 | JX130481 |
| *M.* cf. *simus* **2** W Ecuador | El Oro, Ecuador | TTU 102632 | TK 135274 | JX130456 |
| *M. simus* E Ecuador | Pastaza District, Ecuador | TTU 85090 | TK 104318 | JX130506 |
| ***M. sodalis*** | Vermont, USA | THK 2002-JK-01 | – | AM262337 |
| *M.* sp. **1** Venezuela | Guárico, Venezuela | – | TK 15035 | JN020568 |
| *M.* sp. **1** Venezuela | Guárico, Venezuela | N/A | TK 15036 | JX130451 |
| *M.* sp. **2** E Ecuador | Pastaza District, Ecuador | TTU 85078 | TK 104306 | JX130495 |
| *M.* sp. **2** W Ecuador | Zamora-Chinchipe, Ecuador | N/A | TK 151790 | JX130511 |
| *M.* sp. **2** W Ecuador | Loja, Ecuador | N/A | TK 151860 | JX130512 |
| *M.* sp. **3** W Ecuador | Zamora-Chinchipe, Ecuador | N/A | TK 151772 | JX130507 |
| *M.* sp. **3** W Ecuador | Zamora-Chinchipe, Ecuador | N/A | TK 151823 | JX130508 |
| *M.* sp. **4** W Ecuador | Loja, Ecuador | N/A | TK 151755 | JX130509 |
| *M.* sp. **4** W Ecuador | Loja, Ecuador | N/A | TK 151869 | JX130510 |
| *M.* sp. **5** Mexico | San Luis Potosi, Mexico | TTU 35361 | TK 14521 | JX130518 |
| *M.* sp. **6** Peru | Huánuco Dept., Peru | N/A | TK 22703 | JX130436 |
| *M.* sp. **6** Paraguay | Dept. Canindeyú, Paraguay | N/A | TK 121165 | JX130488 |
| *M.* sp. **7** W Ecuador | Chimborazo, Ecuador | QCAZ 11380 | TK 161638 | JX130516 |
| *M.* sp. **8** W Ecuador | El Oro, Ecuador | TTU 102707 | TK 135095 | JX130471 |
| ***M. thysanodes*** | Brewster Co. Texas, USA | TTU 79327 | TK 78796 | AF376869 |
| *M.* cf. *thysanodes* **1** Mexico | Durango, Mexico | TTU 75557 | TK 48571 | JX130459 |
| *M.* cf. *thysanodes* **1** Mexico | Jalisco, Mexico | TTU 75573 | TK 48589 | JX130460 |
| *M. thysanodes* Mexico | Nuevo Leon, Mexico | TTU 57052 | TK 32509 | JX130458 |
| *M. thysanodes* Mexico | Nuevo Leon, Mexico | TTU 57061 | TK 32526 | JX130487 |
| *M. thysanodes* Mexico | Durango, Mexico | N/A | TK 72301 | JX130461 |
| ***M. velifer*** | Sonora, Mexico | MVZ 146766 | – | AF376870 |
| *M. velifer* El Salvador | Santa Ana, El Salvador | TTU 60983 | TK 34862 | JX130477 |
| *M.* *velifer* Mexico | Tamaulipas, Mexico | TTU 44816 | TK 27026 | JX130478 |
| *M.* *velifer* Mexico | Michoacán, Mexico | UAMI 15304 | TK 45268 | JX130462 |
| *M. velifer* Mexico | Michoacán, Mexico | UAMI 15306 | TK 45271 | JX130438 |
| *M. velifer* Texas | Texas, USA | TTU 109261 | TK 128069 | JX130468 |
| ***M. vivesi*** | Baja California, Mexico | – | – | AJ504407 |
| ***M. volans* A** | Jeff Davis Co. Texas, USA | TTU 7954 | TK 78980 | AF376871 |
| ***M. volans* B** | Jeff Davis Co. Texas, USA | TTU 79518 | TK 78925 | AF376872 |
| ***M. yumanensis*** | California, USA | MVZ 155853 | – | AF376875 |
| *M.* cf. *yumanensis* **1** Mexico | Durango, Mexico | TTU 75621 | TK 48533 | JX130483 |
