# Supplementary material for: Genetic Diversity of Neotropical Myotis (Chiroptera: Vespertilionidae) with an Emphasis on South American Species
Source: PLoS One. 2012 Oct 3;7(10):e46578. doi: 10.1371/journal.pone.0046578 (PMC3463536; doi:10.1371/journal.pone.0046578)
Supplement: Table S2 — Additional specimens sequenced but not used in final genetic analyses. (DOCX) [file pone.0046578.s002.docx]

**Table S2. Additional specimens**. Abbreviations and acronyms for institutions or individual collections are as follows: Cornelio Sanchez-Hernandez (CS-H), Carnegie Museum of Natural History (CM), Jane A. Groen (JAG), Museum of Texas Tech University (TTU = voucher specimen, TK = tissue), Universidad Autonoma Metropolitana-Iztapalapa (UAMI), and University of Nebraska State Museum (UNSM ZM). N/A = Not Available.

| **Name** | **Locality** | **Voucher No.** | **Tissue No.** | **Accession No.** |
| --- | --- | --- | --- | --- |
| *M. albescens* | Guárico, Venezuela | TTU 33396 | TK 15370 | JX130521 |
| *M. albescens* | Pastaza District, Ecuador | TTU 85091 | TK 104319 | JX130522 |
| *M. albescens* | Loja, Ecuador | TTU 103803 | TK 135299 | JX130523 |
| *M. californicus* | Michoacán, Mexico | CS-H | TK 46329 | JX130524 |
| *M. dominicensis* | St. Joseph's Parish, Dominica | TTU 31507 | TK 15619 | JN020555 |
| *M. dominicensis* | St. Joseph's Parish, Dominica | TTU 31508 | TK 15620 | JN020556 |
| *M. keaysi* | Yucatan, Mexico | JAG 286 | TK 13523 | JX130525 |
| *M. keaysi* | San Luis Potosi, Mexico | TTU 35360 | TK 14520 | JX130526 |
| *M. lucifugus* | Michoacán, Mexico | N/A | TK 46331 | JX130527 |
| *M. nigricans* | La Paz, Bolivia | TTU 34953 | TK 14555 | JX130528 |
| *M. nigricans* | Guárico, Venezuela | TTU 48162 | TK 15125 | JX130529 |
| *M. nigricans* | Guárico, Venezuela | N/A | TK 15161 | JX130530 |
| *M. nigricans* | Guárico, Venezuela | TTU 48163 | TK 15186 | JX130531 |
| *M. nigricans* | Guárico, Venezuela | TTU 48164 | TK 15254 | JX130532 |
| *M. nigricans* | Guárico, Venezuela | TTU 48168 | TK 15258 | JX130533 |
| *M. nigricans* | Nickerie, Suriname | CM 77694 | TK 17819 | JX130534 |
| *M. nigricans* | Barinas, Venezuela | CM 78645 | TK 19538 | JX130535 |
| *M. nigricans* | Para, Suriname | CM 77700 | TK 21063 | JX130536 |
| *M. nigricans* | Huánuco Dept., Peru | TTU 46344 | TK 22871 | JX130537 |
| *M. nigricans* | Huánuco Dept., Peru | TTU 46346 | TK 22896 | JX130538 |
| *M. nigricans* | Concepciόn, Paraguay | TTU 99516 | TK 64239 | JX130539 |
| *M. nigricans* | Boquerón, Paraguay | TTU 99151 | TK 65051 | JX130540 |
| *M. nigricans* | Guayas, Ecuador | TTU 103751 | TK 134657 | JX130541 |
| *M. nigricans* | St. Paul Parish, Tobago | UNSM ZM-29483 | TK 157638 | JN020574 |
| *M. nigricans* | Jalisco, Mexico | TTU 110033 | TK 148775 | JX130542 |
| *M. riparius* | Guárico, Venezuela | TTU 48169 | TK 15037 | JX130543 |
| *M. riparius* | Guárico, Venezuela | TTU 48170 | TK 15038 | JX130544 |
| *M. riparius* | Guayas, Ecuador | TTU 103752 | TK 134658 | JX130545 |
| *M. riparius* | El Oro, Ecuador | TTU 102760 | TK 135380 | JX130546 |
| *M. riparius* | El Oro, Ecuador | TTU 102765 | TK 135385 | JX130547 |
| *M. riparius* | El Oro, Ecuador | TTU 102487 | TK 135419 | JX130548 |
| *M. riparius* | El Oro, Ecuador | TTU 102489 | TK 135421 | JX130549 |
| *M. riparius* | El Oro, Ecuador | TTU 102490 | TK 135422 | JX130550 |
| *M. riparius* | El Oro, Ecuador | TTU 102491 | TK 135423 | JX130551 |
| *M. riparius* | El Oro, Ecuador | TTU 102492 | TK 135424 | JX130552 |
| *M. riparius* | El Oro, Ecuador | TTU 102493 | TK 135425 | JX130553 |
| *M. riparius* | El Oro, Ecuador | TTU 102494 | TK 135426 | JX130554 |
| *M. riparius* | El Oro, Ecuador | TTU 102495 | TK 135427 | JX130555 |
| *M. riparius* | El Oro, Ecuador | TTU 102496 | TK 135428 | JX130556 |
| *M. riparius* | El Oro, Ecuador | TTU 102498 | TK 135430 | JX130557 |
| *M. riparius* | El Oro, Ecuador | TTU 102500 | TK 135432 | JX130558 |
| *M. riparius* | El Oro, Ecuador | TTU 102517 | TK 135449 | JX130559 |
| *M. riparius* | El Oro, Ecuador | TTU 102518 | TK 135450 | JX130560 |
| *M. riparius* | El Oro, Ecuador | TTU 102519 | TK 135451 | JX130561 |
| *M. riparius* | El Oro, Ecuador | TTU 102520 | TK 135452 | JX130562 |
| *M. riparius* | El Oro, Ecuador | TTU 102521 | TK 135453 | JX130563 |
| *M. riparius* | El Oro, Ecuador | TTU 102522 | TK 135454 | JX130564 |
| *M. riparius* | El Oro, Ecuador | TTU 102523 | TK 135455 | JX130565 |
| *M. riparius* | El Oro, Ecuador | TTU 102524 | TK 135456 | JX130566 |
| *M. riparius* | El Oro, Ecuador | TTU 102525 | TK 135457 | JX130567 |
| *M. riparius* | El Oro, Ecuador | TTU 102526 | TK 135458 | JX130568 |
| *M. riparius* | El Oro, Ecuador | TTU 102527 | TK 135459 | JX130569 |
| *M. riparius* | El Oro, Ecuador | TTU 102742 | TK 135472 | JX130570 |
| **Name** | **Locality** | **Voucher No.** | **Tissue No.** | **Accession No.** |
| *M. riparius* | El Oro, Ecuador | TTU 102811 | TK 135757 | JX130571 |
| *M. simus* | El Oro, Ecuador | TTU 102681 | TK 135069 | JX130572 |
| *M*. sp. | Guárico, Venezuela | N/A | TK 15067 | JX130573 |
| *M*. sp. | Olancho, Honduras | TTU 84743 | TK 102086 | JX130574 |
| *M.* sp. | Tungurahua Province, Ecuador | TTU 85044 | TK 104272 | JX130575 |
| *M*. sp. | Esmeraldas Province, Ecuador | TTU 85347 | TK 104575 | JX130576 |
| *M*. sp. | Esmeraldas Province, Ecuador | TTU 85411 | TK 104639 | JX130577 |
| *M*. sp. | Guayas, Ecuador | TTU 103671 | TK 134581 | JX130578 |
| *M*. sp. | Guayas, Ecuador | TTU 103501 | TK 134668 | JX130579 |
| *M*. sp. | Guayas, Ecuador | TTU 103490 | TK 135013 | JX130580 |
| *M*. sp. | El Oro, Ecuador | TTU 102775 | TK 135395 | JX130581 |
| *M*. sp. | Tamaulipas, Mexico | TTU 110032 | TK 149757 | JX130582 |
| *M*. sp. | Zamora-Chinchipe, Ecuador | N/A | TK 151765 | JX130583 |
| *M*. sp. | Zamora-Chinchipe, Ecuador | N/A | TK 151786 | JX130584 |
| *M*. sp. | Loja, Ecuador | N/A | TK 151805 | JX130585 |
| *M*. sp. | Zamora-Chinchipe, Ecuador | N/A | TK 151814 | JX130586 |
| *M*. sp. | Loja, Ecuador | N/A | TK 151868 | JX130587 |
| *M. thysanodes* | Michoacán, Mexico | CS-H | TK 47922 | JX130588 |
| *M. velifer* | Michoacán, Mexico | UAMI 15305 | TK 45270 | JX130589 |
| *M. volans* | Texas, USA | TTU 109262 | TK 128076 | JX130590 |
| *M. volans* | Michoacán, Mexico | TTU 104795 | TK 150599 | JX130591 |
| *M. yumanensis* | Michoacán, Mexico | CS-H | TK 46327 | JX130592 |
